# Household animal ownership is associated with infant animal source food consumption in Bangladesh

**DOI:** 10.1111/mcn.13495

**Published:** 2023-03-31

**Authors:** Monica M. Pasqualino, Saijuddin Shaikh, Md Tanvir Islam, Shahnaj Parvin, Hasmot Ali, John McGready, Alain B. Labrique, Md Iqbal Hossain, Amanda C. Palmer

**Affiliations:** ^1^ Department of International Health Johns Hopkins Bloomberg School of Public Health Baltimore Maryland USA; ^2^ The JiVitA Project Gaibandha Bangladesh; ^3^ Department of Biostatistics Johns Hopkins Bloomberg School of Public Health Baltimore Maryland USA; ^4^ icddr,b Dhaka Bangladesh

**Keywords:** complementary feeding, diet, empowerment, gender, infant and child nutrition, livestock, South Asia

## Abstract

Context‐specific research is needed on the relationship between household animal production and nutrition outcomes to inform programmes intervening in small‐scale animal production. We examined associations between household animal/fishpond ownership and animal source food (ASF) consumption among 6‐ to 12‐month‐old infants enroled in the control arm of a cluster‐randomised controlled trial in rural Bangladesh. We measured ASF consumption using a 7‐day food frequency questionnaire at 6, 9 and 12 months and assessed household animal/fishpond ownership at 12 months. We developed negative binomial regression models with random intercepts for infant and cluster, controlling for infant age and sex, maternal age, socioeconomic status and season. Models were stratified by a dichotomised maternal decision‐making score. Compared with infants in households without each animal type, those with 4–10 and ≥11 poultry consumed eggs 1.3 (95% confidence interval [CI]: 1.1, 1.6) and 1.6 (95% CI: 1.3, 2.0) times more, respectively; 2–3 and ≥4 dairy‐producing animals consumed dairy 1.9 (95% CI: 1.3, 2.7) and 2.0 (95% CI: 1.3, 3.1) times more, respectively; and ≥12 meat‐producing animals consumed meat 1.4 (95% CI: 1.0, 1.8) times more. It was unclear whether there was an association between fishpond ownership and fish consumption. Our results did not suggest that maternal decision‐making power was a modifier in the relationship between animal/fishpond ownership and ASF consumption. In this South Asian context, strategies intervening in household animal production may increase infant consumption of eggs, dairy and meat, but not necessarily fish. Research is needed on the role of market access and other dimensions of women's empowerment.

## Key messages

- Household ownership of poultry and dairy‐ and meat‐producing animals was associated with higher infant intakes of eggs, dairy and meat, respectively. There was not a clear association between household fishpond ownership and infant fish consumption.
- While we found no significant differences in these relationships by maternal decision‐making power, future research in this setting should consider the relative importance of other dimensions of women's empowerment or other contextual factors, such as market access.
- In Bangladesh and similar rural South Asian settings, programmes aiming to impact infant and child diet through agricultural interventions must assess the factors constraining both production and consumption.

## 1 INTRODUCTION

Animal source food (ASF) consumption may contribute to improved early growth and cognitive development (Dror & Allen, 2011). In low‐resource settings like rural Bangladesh, ASF is not regularly included in the diets of infants and young children (Campbell et al., 2016; Rah et al., 2010). Numerous programmes in low‐ and middle‐income countries (LMICs) have intervened in household animal production, primarily focusing on reducing poverty and improving livelihoods but also aiming to improve human nutrition (Ruel et al., 2018). Overall, evaluations have shown that these programmes positively impact the production and consumption of targeted foods when designed to address the specific needs and constraints of a population (Darrouzet‐Nardi et al., 2016). Observational studies have demonstrated the importance of considering contextual factors that may influence the relationship between household animal ownership and human nutrition when designing interventions (de Bruyn et al., 2018; Girard et al., 2012; Masset et al., 2012). Country‐ and region‐specific research is needed to better understand how small‐scale animal production relates to ASF consumption among infants and young children.

Agricultural production can potentially impact infant and child nutrition directly through increased consumption of promoted foods or indirectly through increases in income from the sale of agricultural products (Girard et al., 2012; Herforth & Harris, 2014; Turner et al., 2013). These pathways are often affected by factors like socioeconomic status, social norms, seasonality and market access that influence decisions on how food produced at home is utilised (Ruel et al., 2018). In the case of ASF, such decisions can also depend on the type and scale of animal production. For example, a study in Nepal observed that even low levels of household animal ownership were positively associated with egg and dairy intake among children, but meat intake was only higher among children living in households owning a large number of animals typically raised for their meat. This suggests that the effect of small‐scale agricultural strategies on a child diet depends on the targeted animal product (Broaddus‐Shea et al., 2020).

Among the contextual factors considered in studies on agriculture–nutrition linkages, women's empowerment has been a key element. In Bangladesh, where the majority (56%) of employed women work in agriculture, programmes have intervened in farming systems, such as poultry and vegetable production, that are typically managed by women (Nielsen et al., 2003; Shanta et al., 2017; World Bank, 2020). However, few studies in Bangladesh have explored empowerment in terms of women's engagement in household decisions related to food and nutrition (Yosef et al., 2015). Most studies in LMICs on women's empowerment have been conducted in Sub‐Saharan Africa and few have examined the role that gender plays in agriculture–nutrition pathways in South Asia (Akter et al., 2017). This is a significant gap, as gender norms differ across regions, countries and communities.

This study aimed to strengthen the evidence on the relationship between household animal production and infant consumption of animal products to inform future programmes in Bangladesh and similar settings in South Asia. Our objectives were to (1) characterise household ownership of poultry, livestock and fishponds, (2) examine the associations between different types of animal/fishpond ownership and ASF consumption among infants 6–12 months of age, and (3) assess if one dimension of women's empowerment (i.e., decision‐making power) modified these associations in rural Bangladesh.

## 2 METHODS

### 2.1 Setting and study population

Data for this study were collected as part of a cluster‐randomised controlled trial at the JiVitA Research Site located in Gaibandha District in Rangpur Division in northwestern Bangladesh (clinical registration No. NCT03683667). The site has been divided into 566 clusters and has a population of about 630,000. It has hosted several studies of maternal and child health and is representative of national rural infrastructure, maternal and child nutritional status, and health and nutrition services (Christian et al., 2015; Labrique et al., 2011; Merrill et al., 2011; Rah et al., 2009).

### 2.2 Study design

The site operates a pregnancy surveillance system through which women were identified for enrolment in the mCARE‐II trial, which evaluated the effect of a mHealth intervention in antenatal and postnatal care coverage (clinical registration No. NCT02909179). Data on the characteristics of women enroled in the mCARE‐II trial were collected through an interview scheduled at the time of pregnancy identification and included women's age and education, husbands' occupation, and household size and asset ownership.

Infants born to women enroled in the mCARE‐II trial who reached 3 months of age during a 1‐year enrolment period (September 2018–September 2019) were considered eligible for a trial evaluating the independent and combined effects of a protein intervention and enteric pathogen control intervention on linear growth. For this trial, clusters were randomised to presumptive treatment with azithromycin or a placebo at 6 and 9 months of age and to one of three nutrition interventions (provision of a daily egg, a protein‐rich blended food or a control blended food) or the control. Nutrition education was provided to all arms and consisted of age‐specific messages on infant and young child feeding, which included feeding animal products (e.g., fish, egg, liver) daily with *dal*, leafy greens and fruits/vegetables, as well as on health and hygiene. These messages, which were adapted from Alive and Thrive modules, were standardised and provided to caregivers once a month by field distributors in the form of audio recordings and illustrative pamphlets (Alive and Thrive 2020; Menon et al., 2016). The present analysis is restricted to subjects in the control arm, irrespective of the enteric pathogen control intervention, as they did not receive food provided by the trial.

Protocols were approved by the Institutional Review Board of the Johns Hopkins Bloomberg School of Public Health (Baltimore, MD) and the Research and Ethics Review Committee of the International Center for Diarrhoeal Disease Research, Bangladesh (Dhaka, Bangladesh).

### 2.3 Sample size

The trial was designed based on the primary linear growth outcome. A 1‐year enrolment period was anticipated to yield a cohort of 3180 infants, which would enable the detection of a difference in the mean length‐for‐age *z*‐score (LAZ) of 0.165 between groups at 12 months with 80% power. However, the pregnancy surveillance system registered approximately 30% more pregnancies than anticipated. We attributed this to an inaccurate assumption that fertility would decline based on data from the period during which pregnancies were last registered at the site (2008–2012). Based on actual births, enrolment at 3 months, and an estimated 10% loss to follow‐up, we modified the enrolment period from 1 year to 9 months to yield a cohort of about 5400 subjects. We anticipated that a quarter of this cohort (about 1400 subjects) would receive the control. For the current study, which involved longitudinal data with discrete outcome variables, we ran a simulation analysis to ensure that the available sample size was adequate to detect a meaningful difference in outcomes between households with and without animal ownership with 80% power and *α* = 0.05. We referred to the literature to inform assumptions on within‐subject variation, cluster‐level variability and estimated differences in outcomes as data on the frequency of ASF consumption among children in the study area were not available (Arnold et al., 2011; Broaddus‐Shea at al., 2020; Darrouzet‐Nardi et al., 2016; Diggle et al., 2002; Murty et al., 2016).

### 2.4 Data collection

Household consent (3‐month) visits began in September 2018, with 6‐month visits and intervention delivery beginning in January 2019. Household 12‐month visits were scheduled through April 2020 but suspended at the onset of COVID‐19 in March 2020. For the remaining cases, field workers collected data over the telephone. Field interviewers visited households when infants reached approximately 6, 9 and 12 months of age to administer a questionnaire to the primary caregiver and measure infant size. Household food insecurity (HFI) was measured at the 6‐month visit using a nine‐item questionnaire (Na et al., 2016). Infant weight and length were measured using standard protocols and breastfeeding status was assessed by asking if the infant was currently being breastfed (de Onis et al., 2004). Seven‐day frequency of ASF consumption was assessed at each visit based on a prespecified list of items that included cow, goat, sheep, or buffalo milk; yogurt or other dairies; chicken egg; duck egg; fresh fish; dried fish; chicken, duck, or goose flesh meat; goat or lamb flesh meat; beef or water buffalo flesh meat; and liver. For each ASF listed, the interviewer asked, ‘In the past week, that is from last (today's day) through yesterday's night, how many times has your child eaten (the item)?’ After completing the listed items, the interviewer asked if the infant had eaten any additional animal products.

Household poultry, livestock and fishpond ownership was measured at 12 months. The interviewer first asked the respondent if the household had owned any of the following in the past 6 months: (1) chickens, (2) ducks, (3) cows or water buffalo, (4) goats or sheep, or (5) fishponds. For each item, if the respondent answered yes, the interviewer proceeded by asking, ‘In the past 30 days, that is since (today's date) of (last month) until last night how many [animal type/fishponds] have been in your household?’ The interviewer also asked if the animals and fishponds were male‐owned, female‐owned, or male and female co‐owned. To collect data on the production and utilisation of animal products, the respondent was asked to specify the number of live animals sold and the amount of animal products (e.g., eggs, milk) produced, consumed and sold by the household in the past month. Maternal decision‐making related to food and health was assessed at the 12‐month visit using an eight‐question module that had been previously developed and implemented in the study area. The interviewer asked the respondent how often she was able to decide to buy: (1) small amounts of food like rice, vegetables, *dal* and fish, (2) larger amounts of food like a bag of rice, (3) clothes for herself, (4) medicine for herself, (5) toilet articles for herself like soap, toothpaste, (6) clothes for children, (7) medicine for children, and (8) special food for children. Response options included never, sometimes, and always/often and were coded as zero, one, and two, respectively.

### 2.5 Statistical analysis

Baseline characteristics were summarised as mean ± SD for continuous variables and the number of subjects and percentages for binary and categorical variables. LAZ, weight‐for‐length *z*‐scores (WLZ) and weight‐for‐age *z*‐scores (WAZ) were derived using WHO Child Growth Standards and used to classify infants as stunted (LAZ < −2), wasted (WLZ < −2) or underweight (WAZ < −2) (WHO Multicentre Growth Reference Study Group, 2006). A Living Standards Index (LSI) was created to characterise socioeconomic status using principal components analysis based on household assets and dwelling characteristics and categorised into quintiles (Gunnsteinsson et al., 2010). The LSI did not include ownership of animals or fishponds. HFI was categorised as none (HFI = 9), mild (HFI > 9 to HFI < 16) and severe (HFI ≥ 16) based on an examination of the distribution.

Outcome variables included the number of times an infant consumed egg (chicken or duck), dairy (milk or yogurt), meat (flesh meat or liver from poultry, goats, sheep, cows or water buffalo), or fish (dried or fresh) in the last 7 days. To generate each outcome variable, the frequencies of consumption for the relevant food items were summed at each timepoint. Exposure variables included the number of animals or fishponds owned by a household. For livestock ownership, animal types were grouped together based on the animal product for which they were commonly raised, thereby creating three variables: (1) poultry (chicken and duck), (2) dairy‐producing animals (goats, sheep, cows and water buffalo), and (3) meat‐producing animals (poultry, goats, sheep, cows and water buffalo).

We explored the distributions of each exposure variable to categorise them into levels of ownership. The cut‐offs for low, medium and high animal ownership were based on the 25th, 50th and 75th percentiles of the distributions of each variable, respectively, among households owning any animals. For fishponds, the cut‐off for a medium level of ownership was ≤75th percentile of the distribution and >75th percentile for a high level of ownership, among households with any fishponds. Poultry ownership was categorised as none, 1–3 poultry (low), 4–10 poultry (medium), or ≥11 poultry (high); ownership of dairy‐producing animals as none, 1 animal (low), 2–3 animals (medium), or ≥4 animals (high); ownership of meat‐producing animals as none, 1–4 animals (low), 5–11 animals (medium), or ≥12 animals (high); and fishpond ownership as none, 1 pond (medium), or ≥2 ponds (high). We stratified the number of animals/fishponds owned by season (i.e., monsoon, winter, summer) and gender to explore variations in ownership. Responses from the eight‐item maternal decision‐making questionnaire were used to generate a score ranging from 0 to 16, with higher values indicating greater decision‐making power. After exploring the distribution, the score was dichotomised around the median to categorise respondents as having either ‘low’ or ‘high’ decision‐making power.

Trends on average consumption and variance were examined for each outcome variable to inform model assumptions. Four separate regression models were developed to assess associations between poultry ownership and frequency of infants' egg intake, ownership of dairy‐producing animals and dairy intake, ownership of meat‐producing animals and meat intake, and fishpond ownership and fish intake. Each model was specified as a negative binomial regression model with random intercepts for infant and cluster and adjusted for infant age and sex, maternal age, LSI, and season (Diggle et al., 2002; Hilbe, 2011). Each adjusted model was then stratified by the dichotomous maternal decision‐making power variable to determine if decision‐making power modified the relationship. Effect estimates were calculated for each stratum of decision‐making power and a ratio of ratios was estimated to test for the significance of modification on a multiplicative scale (Knol & VanderWeele, 2012).

We explored reasons for missingness and implemented multiple imputations by chained equations (*n* = 35) to impute missing values for the exposure variables and covariates. For the outcome variables, all available data were included in the analyses. Sensitivity analyses using subjects with complete data were conducted to compare findings with those from the approach using all available outcome data. All analyses were performed using Stata version 14.2 (StataCorp LP). *p* < 0.05 were considered statistically significant.

## 3 RESULTS

In total, 1698 infants from 141 clusters were eligible for enrolment into the control arm of the trial. Parental consent was obtained for 1511 infants (90.0%). Data were available on any outcome variable for 1467 infants (Supporting Information: Figure 1). Among these, 30.5% were missing data on at least one outcome variable across timepoints and 12.4% were missing data on at least one exposure variable or covariate. The percentage of subjects missing outcome data was similar at each timepoint. Missing data was primarily due to failure to complete an interview before subjects aged out of eligibility for that interview. Subjects with complete data and those missing data differed on a number of baseline characteristics (Supporting Information: Table 1). At baseline, infants were a mean age of 6.3 ± 0.3 months (Table 1). Prevalences of stunting, wasting and underweight were 19.3%, 5.6% and 17.8%, respectively. Almost all infants had been breastfed in the last 24 h (98.1%). Most infants had mothers with primary education (71.7%) and fathers who worked as labourers (35.5%) or business owners (33.5%). The average household size was 4.5 ± 2.0.

**Table 1:** Household, parental and infant baseline characteristics of infants enroled in a longitudinal observational study nested within a trial providing a 6‐month protein intervention in rural Bangladesh (*N* = 1467)^a^.

Characteristic	*n*	% or mean ± SD
Household		
Household size	1449	4.5 ± 2.0
LSI quintile		
1st (lowest)	1449	20.6
2nd		19.5
3rd		20.5
4th		19.8
5th (highest)		19.6
HFI		
None	1276	68.8
Mild		28.9
Severe		2.4
Parental		
Maternal age, year	1426	23.5 ± 5.4
Maternal education		
No schooling	1447	9.9
1–9 years		71.7
SSC passed		5.6
≥11 years		12.8
Paternal occupation		
Farmer/sharecropper	1271	12.1
Labourer		35.5
Own business		33.5
Private/government service		18.1
Other		0.8
Infant		
Age, month	1296	6.3 ± 0.3
Sex, M	1467	52.4
Stunting, LAZ < −2	1280	19.3
Wasting, WLZ < −2	1267	5.6
Underweight, WAZ < −2	1280	17.8
Breastfed, last 24 h	1296	98.1

Abbreviations: HFI, household food insecurity; LAZ, length‐for‐age *z*‐score; LSI, Living Standards Index; SSC, secondary school certificate; WAZ, weight‐for‐age *z*‐score; WLZ, weight‐for‐length *z*‐score.

^a^ LSI calculated based on household assets and dwelling characteristics using principal components analysis; HFI estimated using a nine‐item questionnaire collapsed into an index with possible scores ranging from 9 to 36 and categorised as none (HFI = 9), mild (HFI > 9 to HFI < 16) and severe (HFI ≥ 16);

Most households owned at least one animal: 75.2% owned poultry, 67.1% owned dairy‐producing animals and 86.7% owned animals typically raised for their meat (Table 2). Less than a quarter of households (22.7%) owned fishponds. Few households owned a high number of poultry (≥11 poultry, 13.0%), dairy‐producing animals (≥4 animals, 17.7%), meat‐producing animals (≥12 animals, 19.0%) or fishponds (≥2 ponds, 3.9%). Animal production was greater with higher levels of ownership (e.g., households with 1–3, 4–9 and ≥11 poultry produced a median of 4, 12 and 25 eggs in the past month, respectively); however, it was low overall. The median number of animals/fishponds owned was relatively stable across seasons (Supporting Information: Table 2). Poultry were primarily managed by women (about 85.0% female‐owned vs. 4.0% male‐owned), cattle and fishponds by men (56.4% male‐owned vs. 13.5% female‐owned and 81.6% male‐owned vs. 3.3% female‐owned, respectively), and ownership of goats and sheep was relatively equivalent between women and men (47.3% female‐owned, 27.5% male‐owned and 25.2% female/male co‐owned).

**Table 2:** Household production and utilisation of animal products in the last 30 days, by household animal and fishpond ownership, in a longitudinal observational study nested within a trial providing a 6‐month protein intervention to infants in rural Bangladesh (*N* = 1467)^a^.

	Owned		Median (25th, 75th percentile)		
Ownership type	*n*	%	Amount produced	Amount sold	Amount consumed
Poultry			Eggs (No.)		
No poultry	1335	24.8	–	–	–
Low (1–3 poultry)		26.1	4 (0, 12)	0 (0, 0)	0 (0, 6)
Medium (4–10 poultry)		36.1	12 (2, 29)	0 (0, 0)	4 (0, 15)
High (≥11 poultry)		13.0	25 (10, 51)	0 (0, 8)	12 (0, 26)
Dairy‐producing animals			Milk (kg)		
No animals	1335	32.9	–	–	–
Low (1 animal)		18.1	0 (0, 0)	0 (0, 0)	0 (0, 0)
Medium (2–3 animals)		31.3	0 (0, 0)	0 (0, 0)	0 (0, 0)
High (≥4 animals)		17.7	0 (0, 15)	0 (0, 0)	0 (0, 15)
Meat‐producing animals			Animals (No.)		Meat (kg)
No animals	1333	13.3	–	–	–
Low (1–4 animals)		31.5	3 (2, 4)	0 (0, 0)	0 (0, 0)
Medium (5–11 animals)		36.2	7 (6, 9)	0 (0, 0)	0 (0, 0)
High (≥12 animals)		19.0	16 (13, 22)	0 (0, 2)	1 (0, 2)
Fishponds			Fish (kg)		
No ponds	1323	77.3	–	–	–
Medium (1 pond)		18.8	3 (0, 10)	0 (0, 0)	2 (0, 5)
High (≥2 ponds)		3.9	7 (1, 30)	0 (0, 0)	5 (1, 15)

^a^ Poultry include chickens and ducks; dairy‐producing animals include cows, water buffalo, goats and sheep; meat‐producing animals include poultry, cows, water buffalo, goats and sheep. For meat‐producing animals, the amount produced is the number of animals owned; the amount sold is expressed in the number of animals because they are typically sold live; the amount consumed is expressed in meat (kg) because they are typically slaughtered for home consumption.

Overall, infant consumption of ASF was low and increased with age (Table 3). At 6 months of age, most infants had not consumed any ASF in the past 7 days. Consumption of each animal product increased at 9 months, particularly of fish. At 12 months of age, 68.1% of infants had consumed fish in the past 7 days, 54.2% had consumed eggs, 48.3% had consumed meat and 29.1% had consumed dairy. The median frequency of egg and fish intake in the last week was once and twice, respectively, and that of dairy or meat was zero times.

**Table 3:** Frequency of animal source food consumption in the last 7 days among infants enroled in a longitudinal observational study nested within a trial providing a 6‐month protein intervention in rural Bangladesh, by age (*N* = 1467).

	6 month		9 month		12 month	
	*n* = 1260		*n* = 1300		*n* = 1296	
Animal source food^a^	Any, %	Median (25th, 75th percentiles)	Any, %	Median (25th, 75th percentiles)	Any, %	Median (25th, 75th percentiles)
Eggs						1 (0, 3)
Dairy	14.1	0 (0, 0)	23.2	0 (0, 0)	29.1	0 (0, 1)
Meat	22.1	0 (0, 0)	40.6	0 (0, 1)	48.3	0 (0, 2)
Fish	19.0	0 (0, 0)	60.2	1 (0, 3)	68.1	2 (0, 4)

^a^ Eggs include chicken and duck eggs; dairy includes animal milk and yogurt; meat includes flesh meat and liver from poultry, cattle and goats/sheep; fish includes fresh and dried fish.

In adjusted analyses, compared with infants in households without poultry, those with medium (4–10 poultry) and high (≥11 poultry) levels of ownership consumed eggs 1.3 (95% confidence interval [CI]: 1.1, 1.6) and 1.6 (95% CI: 1.3, 2.0) times more, respectively, in the last 7 days. Compared with those without dairy‐producing animals, those with medium (2–3 dairy‐producing animals) and high (≥4 dairy‐producing animals) levels of ownership consumed dairy 1.9 (95% CI: 1.3, 2.7) and 2.0 (95% CI: 1.3, 3.1) times more, respectively; and compared with those without meat‐producing animals, those with a high level of ownership (≥12 meat‐producing animals) consumed meat 1.4 (95% CI: 1.0, 1.8) times more. It was not certain whether there was an association between household fishpond ownership and infant fish intake (Table 4). There was no consistent evidence suggesting that maternal decision‐making power significantly modified the relationship between household animal production and infant consumption of ASF. For each level of animal/fishpond ownership, the estimated rate of weekly intake of the respective animal product compared to the rate when no animals/fishponds were owned was statistically similar between mothers reporting low and high decision‐making power, with an exception of those living in households with a high number of dairy‐producing animals (Table 5). Sensitivity analyses using a complete case analysis approach resulted in similar findings (Supporting Information: Tables 3 and 4).

**Table 4:** Associations between household animal and fishpond ownership and animal source food consumption were observed among infants at three timepoints (6, 9 and 12 months of age) who were enroled in a longitudinal observational study nested within a 6‐month protein supplementation trial in rural Bangladesh (*N* = 1467)^a^.

	7‐day frequency of egg intake		Ownership of dairy‐producing animals	7‐day frequency of dairy intake		Ownership of meat‐producing animals	7‐day frequency of meat intake			7‐day frequency of fish intake	
Poultry ownership	Unadjusted	Adjusted		Unadjusted	Adjusted		Unadjusted	Adjusted	Fish pond ownership	Unadjusted	Adjusted
	IRR (95% CI)	IRR (95% CI)		IRR (95% CI)	IRR (95% CI)		IRR (95% CI)	IRR (95% CI)		IRR (95% CI)	IRR (95% CI)
No poultry	1.00	1.00	No animals	1.00	1.00	No animals	1.00	1.00	No ponds	1.00	1.00
Low (1–3 poultry)	1.10 (0.92, 1.32)	1.15 (0.93, 1.41)	Low (1 animal)	1.33 (0.89, 1.99)	1.35 (0.89, 2.04)	Low (1–4 animals)	1.04 (0.82, 1.32)	1.06 (0.84, 1.35)	–	–	–
Medium (4–10 poultry)	1.31 (1.11, 1.55)**	1.33 (1.10, 1.61)*	Medium (2–3 animals)	1.89 (1.34, 2.66)***	1.87 (1.31, 2.66)***	Medium (5–11 animals)	1.20 (0.95, 1.52)	1.17 (0.93, 1.48)	Medium (1 pond)	1.09 (0.94, 1.26)	1.04 (0.88, 1.23)
High (≥11 poultry)	1.61 (1.30, 1.99)***	1.61 (1.27, 2.04)***	High (≥4 animals)	2.48 (1.67, 3.68)***	2.03 (1.34, 3.09)**	High (≥12 animals)	1.56 (1.21, 2.02)**	1.35 (1.04, 1.75)**	High (≥2 ponds)	1.29 (0.97, 1.73)*	1.08 (0.77, 1.52)

Abbreviation: IRR, incidence rate ratio.

^a^ Negative binomial regression models with random intercepts specified for infant and cluster were used to assess associations following multiple imputations with chained equations (*n* = 35) to impute values for missing exposure variables and covariates. Variables included in adjusted models were infant age and sex, maternal age, socioecono'mic status and season. All available data on the outcome variables were included in the analyses. Poultry included chickens and ducks; dairy‐producing animals included cows, water buffalo, goats and sheep; and meat‐producing animals included poultry, cattle and goats/sheep. Egg intake included that of chicken and duck eggs; dairy included animal milk and yogurt; meat included flesh meat and liver from poultry, cattle and goats/sheep; and fish included fresh and dried fish.

* *p* < 0.01

** *p* < 0.05

*** *p* < 0.001.

**Table 5:** Associations between household animal and fishpond ownership and animal source food consumption among infants at three timepoints (6, 9 and 12 months of age) who were enroled in a longitudinal observational study nested within a trial providing a 6‐month protein intervention in rural Bangladesh, stratified by maternal decision‐making power (*N* = 1467)^a^.

	Frequency of intake in the 7 days before interview		
Animal/fishpond ownership	Low decision‐making power	High decision‐making power	
	IRR (95% CI)	IRR (95% CI)	IRR/IRR
Poultry ownership			
No poultry	1.00	1.00	–
Low (1–3 poultry)	1.27 (0.95, 1.69)	1.06 (0.80, 1.40)	0.83 (0.56, 1.24)
Medium (4–10 poultry)	1.42 (1.08, 1.86)**	1.26 (0.98, 1.62)*	0.89 (0.62, 1.28)
High (≥11 poultry)	1.52 (1.08, 2.13)**	1.74 (1.26, 2.41)**	1.15 (0.72, 1.83)
Ownership of dairy‐producing animals			
No animals	1.00	1.00	–
Low (1 animal)	1.84 (1.01, 3.38)**	1.05 (0.60, 1.82)	0.57 (0.25, 1.28)
Medium (2–3 animals)	2.23 (1.35, 3.69)**	1.63 (1.01, 2.64)**	0.73 (0.37, 1.45)
High (≥4 animals)	3.40 (1.90, 6.11)***	1.24 (0.69, 2.21)	0.36 (0.16, 0.82)**
Ownership of meat‐producing animals			
No animals	1.00	1.00	–
Low (1–4 animals)	1.05 (0.75, 1.48)	1.07 (0.77, 1.48)	1.02 (0.63, 1.63)
Medium (5–11 animals)	1.14 (0.82, 1.60)	1.20 (0.87, 1.65)	1.05 (0.66, 1.67)
High (≥12 animals)	1.33 (0.92, 1.92)	1.37 (1.04, 1.96)*	1.03 (0.63, 1.70)
Fishpond ownership			
No ponds	1.00	1.00	–
Medium (1 pond)	1.08 (0.85, 1.37)	1.00 (0.80, 1.25)	0.93 (0.68, 1.27)
High (≥2 ponds)	1.33 (0.87, 2.03)	0.84 (0.50, 1.42)	0.64 (0.33, 1.23)

Abbreviations: IRR, incidence rate ratio; IRR/IRR, ratio of incidence rate ratios.

^a^ Negative binomial regression models with random intercepts for infant and cluster were specified following multiple imputations with chained equations (*n* = 35) to impute values for missing exposure variables and covariates. Each model was adjusted for infant age and sex, maternal age, socioeconomic status and season. All available data on the outcome variables were included in the analyses. Low decision‐making power was defined as a score from 0 to 8 and high as a score from 9 to 16. Poultry included chickens and ducks; dairy‐producing animals included cows, water buffalo, goats and sheep; and meat‐producing animals included poultries, cattle and goats/sheep. Egg intake included that of chicken and duck eggs; dairy included animal milk and yogurt; meat included flesh meat and liver from poultries, cattle and goats/sheep; and fish included fresh and dried fish. The IRR/IRR is an estimate testing for the significance of effect measure modification on a multiplicative scale; it assessed if the magnitude of the measure of the association (IRR) between animal/fishpond ownership and animal source food consumption significantly differed between infants with mothers who have high decision‐making power versus those with low decision‐making power. If IRR/IRR > 1.0, the magnitude of the rate of animal source food consumption between infants living in households with animals/fishponds versus those without animals/fishponds was greater among infants who have mothers with high decision‐making power compared to those with low decision‐making power; IRR/IRR < 1.0, the magnitude was lower; IRR/IRR = 1.0, there was no difference in magnitude.

* *p* < 0.01

** *p* < 0.05

*** *p* < 0.001.

## 4 DISCUSSION

In this rural setting in Bangladesh, household ownership of poultry and dairy‐ and meat‐producing animals was associated with higher intakes of eggs, dairy and meat among infants 6–12 months of age, respectively; there was not a clear relationship between ownership of fishponds and fish intake. Our results did not suggest that maternal decision‐making power related to nutrition and health inputs was a modifier in the relationship between animal/fishpond ownership and infant consumption of animal products. Despite observing significant associations between household animal ownership and infant intake of animal products, consumption of eggs, dairy products, meat and fish was low. This illustrates the need for programmes to identify the specific constraints to both production and consumption of animal products when designing agricultural‐based interventions aiming to improve early nutrition in South Asia.

Our finding that poultry ownership was positively associated with infants' egg intake is informative given mixed findings from other studies. Small‐scale poultry production is commonly practiced in low‐resource settings because it requires low amounts of inputs and provides potential for income generation and access to ASF (de Bruyn et al., 2015). Studies conducted in Africa have observed that smallholder farmers prioritise the hatching of eggs for replacement stock over home consumption due to high flock mortality and limited egg production. As such, a consistent relationship between poultry‐raising and egg intake among children has not been observed in these settings (de Bruyn et al., 2018; Dumas et al., 2016). In contrast, a study in rural Nepal observed a positive association between poultry ownership and children's egg consumption, which we similarly observed in rural Bangladesh (Broaddus‐Shea et al., 2020). However, differences were found between these two rural South Asian contexts—in Nepal, any level of poultry ownership increased egg intake, with no greater increase observed with higher levels of ownership. The researchers posited that households began selling eggs once they reached higher levels of production. In our study, households owning a high number of poultries maintained a higher rate of egg consumption among infants. Such differences in findings demonstrate how context‐specific factors may influence how farmers utilise poultry and eggs.

Overall, our findings on poultry ownership are reflective of the Bangladeshi context, where poultry raising is a common practice typically managed by women. Programmes supporting small‐scale production have been implemented for decades, and previous studies have shown most households raising poultry set aside eggs for both consumption and sale, with one study estimating that annual median family consumption of eggs was one‐fifth of total eggs produced (Nielsen et al., 2003; Shanta et al., 2017). Our observation of overall small flock sizes and low egg production appears typical of this setting and may explain why the effect we observed on egg consumption at even the highest level of ownership was relatively small (Sarkar & Golam, 2009). These findings suggest that efforts to improve production would benefit infant and child nutrition. However, the low levels of egg production and consumption we observed highlight the challenges that programmes intervening in poultry‐raising may face as well as the need to learn from innovative strategies that have addressed the specific constraints of a population's poultry‐raising system. Strategies have included establishing small‐scale egg production centres that provide training on poultry health and business management; giving high‐yield breeds to farmers; and integrating nutrition‐focused behaviour change communication within production interventions (Dumas et al., 2018; Passarelli et al., 2020). Such approaches must also address poultry‐handling practices that may increase human exposure to zoonotic diseases and pathogens (Harvey et al., 2003).

There is evidence from observational studies and programmatic interventions that ownership of dairy‐producing animals is associated with children's dairy intake, which is consistent with our findings. However, most studies have been undertaken in East Africa where ownership of cattle and goats is relatively common (Ayele & Peacock, 2003; Hoddinott et al., 2015; Kabunga et al., 2017). One study in Bangladesh using nationally representative survey data found that milk consumption was higher among children under 5 years of age living in households with milk‐producing cows (Choudhury & Headey, 2018). It is uncertain, however, how these findings can be applied to policy or programme recommendations in Bangladesh. Livestock production is constrained due to high human population density and lack of available land, resulting in low levels of milk production and a reliance on milk powder imports (Headey & Hoddinott, 2016). Focusing on smaller ruminants that require fewer inputs and land, such as goats and sheep, may be an option to improve household access to dairy. Research would be needed to identify the factors influencing the production and consumption of dairy products from such ruminants.

The association between ownership of animals typically raised for their meat and infants' meat intake was surprising. Considering the low number of animals owned, it would appear unlikely that a household would slaughter poultry or livestock that can be used as a source of eggs or milk or kept as an asset. However, the rate we observed was small and only at a level of high ownership, which is consistent with these practices. Programmes aiming to increase infants' meat consumption would likely need to target income generation and market access (Broaddus‐Shea et al., 2020). It would also be informative to evaluate if larger livestock are raised for agricultural labour or other purposes as this would generate a better understanding of the pathway between productivity, income, and market purchase of ASF and other foods. Several studies have shown that market access influences the association between small‐scale agricultural production and dietary diversity (Jones, 2017; Koppmair & Qaim, 2017; Sibhatu et al., 2015). Rural households often purchase foods from markets, particularly items with higher economic and nutritional value like animal products. Good market access also allows farmers to profit from food items in which they specialise, leading to increases in income and the ability to purchase nutrient‐rich foods that are typically more expensive (Sibhatu & Qaim, 2018). Understanding the infrastructure of market systems is therefore critical to determine how best to capitalise on the trade and consumption of animal products for improved infant and child nutrition.

Fish was the most commonly consumed ASF among infants in our study, which was expected given that numerous national surveys have reported fish to be frequently consumed by households in Bangladesh across social strata (Toufique & Belton, 2014). However, our findings are unclear as to whether there was an association between fishpond ownership and infants' fish intake. Among households with fishponds, most owned one pond and median monthly fish production was relatively low. It is possible that the level of farmed‐fish production was not sufficient to lead to a significant increase in infants' intakes or it was more economical for the household to purchase fish for consumption. Overall, evaluations of aquaculture programmes in Bangladesh have not found an impact on household fish consumption, concluding that households prefer to sell farmed fish for income (Bouis, 2000; Roos et al., 2003; Thompson et al., 2000).

Additionally, fishponds were primarily owned by men in our study. It is possible that animal production managed by women, rather than men, is more likely to be utilised for the benefit of infant and child nutrition. This may provide additional insight into why we observed egg intake to be associated with poultry ownership, which is primarily managed by women, and dairy and meat intake to be associated with ownership of dairy‐ and meat‐producing animals, which include goats that women manage alone or with men. Studies have shown that women are more likely to invest in food and nutrition for children and the family (Handa, 1996; Kennedy & Peters, 1992). When women own livestock, they have greater control over how to use the animal products and the income that comes from them (Salomon, 2015). One study in Kenya, for example, found that female ownership and female/male co‐ownership of livestock were positively associated with child ASF consumption, HAZ, and WAZ, while male ownership of livestocks was not (Jin & Iannotti, 2014). These findings support strategies to increase women's agricultural productivity.

Although our findings did not suggest that maternal decision‐making power modified the relationship between small‐scale animal production and infant diet, it is possible that other components of women's empowerment play roles in this setting. Measuring dimensions of empowerment separately is necessary to better understand which dimensions affect which nutrition outcomes and how these relationships differ by sociocultural context (Ruel et al., 2018). Few studies have examined the role that gender plays in agriculture‐nutrition pathways in Asia. Our study expanded evidence on cross‐cultural variations in this multidimensional construct and highlighted the need for additional research on gender norms in the South Asian context. Other areas of gender equity, such as women's access to and control over income, land, capital, technology, and marketing and their roles in agricultural and nonagricultural organisations, should be further explored in this context (Akter et al., 2017). For example, one study using national survey data in Bangladesh found the Women's Empowerment in Agriculture Index, which measures women's engagement in the agricultural sector, to be positively associated with household dietary diversity (Malapit et al., 2014; Sraboni & Quisumbing, 2018).

Our study had a number of strengths and limitations. As an observational study, there was potential for unmeasured confounding, and it was not possible to determine a causal relationship between animal/fishpond ownership and ASF consumption. The next step would be a randomised controlled trial assessing the impact of an agricultural intervention on infant and child diet in this setting to determine causality. Dietary intake was not directly observed. The nutrition education provided included messaging on feeding ASF to infants, which may have influenced caregivers to over‐report consumption frequency. However, our interviewers were highly trained and had prior experience objectively assessing infant diet using 7‐day food frequency questionnaires (Na et al., 2016). We assessed infant diet at multiple timepoints, which allowed us to capture variations in ASF consumption by age. We assessed household animal production only at 12 months. The number of animals and fishponds owned by a household can vary over time due to changes in season, climate, disease, holidays and other factors. We stratified ownership by season to gain a sense of change in ownership over time and did not observe large differences, leading us to feel confident that the data represented typical ownership patterns. Our questionnaire did not assess the sex or varieties of animals raised, for example, if poultry were layers or broilers, which would have allowed for greater specificity in characterising animal ownership and production.

In this rural setting in Bangladesh, strategies intervening in the production of poultry and dairy‐ and meat‐producing animals may increase intake of eggs, dairy and meat during the early complementary feeding period. It is critical that programmes consider the context‐specific barriers to ASF consumption. These often include limited availability and access, which can be addressed through agricultural or income‐based strategies. They may also include social norms and perceptions about the appropriateness of ASF for infants and children, such as concerns about ingesting meat with a harder texture or fish with bones, which would require behaviour‐focused approaches (Pachón et al., 2007; Thorne‐Lyman et al., 2017). The potential negative externalities of household animal production, such as increased exposure to pathogens and zoonotic disease, should also be addressed. Finally, research is needed in this setting to explore the role of other dimensions of women's empowerment and contextual factors, such as market access, that may influence the relationship between small‐scale animal production and infant and child nutrition.

## AUTHOR CONTRIBUTIONS

Monica M. Pasqualino, Amanda C. Palmer, Alain B. Labrique, and Md Iqbal Hossain designed the research; Saijuddin Shaikh, Md Tanvir Islam, Shahnaj Parvin, and Hasmot Ali conducted the research; Monica M. Pasqualino and John McGready analyzed the data; MMP wrote the paper; and MMP and Amanda C. Palmer had primary responsibility for final content. All authors read and approved the final manuscript.

## CONFLICT OF INTEREST STATEMENT

The authors declare no conflict of interest.

## ETHICS STATEMENT

Protocols were approved by the Institutional Review Board of the Johns Hopkins Bloomberg School of Public Health (Baltimore, MD) and the Research and Ethics Review Committees of the International Center for Diarrhoeal Disease Research, Bangladesh (Dhaka, Bangladesh).

## Supporting information

Supporting information.

## Data Availability

Data described in the manuscript, code book, and analytic code will be made available upon request pending application and approval.
