# Supplementary material for: Household animal ownership is associated with infant animal source food consumption in Bangladesh
Source: Matern Child Nutr. 2023 Mar 31;19(3):e13495. doi: 10.1111/mcn.13495 (PMC10262903; doi:10.1111/mcn.13495)
Supplement: Supplementary file 1 — Supporting information. [file MCN-19-e13495-s001.docx]

| **Supplemental Table 1.** Comparison of household, parental, and infant baseline characteristics between infants with complete data and those missing data enrolled in a longitudinal observational study nested within a trial providing a six-month protein intervention in rural Bangladesh (N=1467)^1^ | | | | |
| --- | --- | --- | --- | --- |
|  | Complete (N=953) | | Missing (N=514) | |
| Characteristic | *n* | % or | *n* | % or |
|  |  | Mean ± SD |  | Mean ± SD |
| Household |  |  |  |  |
| Household size** | 953 | 4.4 ± 2.0 | 496 | 4.7 ± 2.1 |
| LSI quintile** |  |  |  |  |
| 1st (lowest) | 953 | 21.3 | 496 | 19.2 |
| 2nd |  | 19.6 |  | 19.4 |
| 3rd |  | 21.5 |  | 18.5 |
| 4th |  | 20.0 |  | 19.4 |
| 5th (highest) |  | 17.5 |  | 23.6 |
| HFI |  |  |  |  |
| None | 935 | 68.7 | 341 | 69.2 |
| Mild |  | 28.9 |  | 28.4 |
| Severe |  | 2.5 |  | 2.3 |
| Parental and household |  |  |  |  |
| Maternal age, y** | 953 | 23.7 ± 5.4 | 473 | 23.0 ± 5.2 |
| Maternal education** |  |  |  |  |
| No schooling | 952 | 10.1 | 495 | 9.5 |
| 1-9 y |  | 73.7 |  | 67.9 |
| SSC passed |  | 5.9 |  | 5.1 |
| ≥11 y |  | 10.3 |  | 17.6 |
| Paternal occupation** |  |  |  |  |
| Farmer/fisherman | 839 | 10.8 | 432 | 14.6 |
| Laborer |  | 36.1 |  | 34.3 |
| Own business |  | 35.9 |  | 28.9 |
| Private/government service |  | 16.7 |  | 20.8 |
| Other |  | 0.5 |  | 1.4 |
| Infant |  |  |  |  |
| Age, mo** | 953 | 6.3 ± 0.3 | 343 | 6.4 ± 0.3 |
| Sex, M** | 953 | 49.8 | 514 | 57.2 |
| Stunting, LAZ <-2 | 943 | 19.5 | 337 | 18.7 |
| Wasting, WLZ <-2 | 934 | 5.0 | 333 | 7.2 |
| Underweight, WAZ <-2 | 942 | 17.1 | 338 | 19.8 |
| Breastfed, last 24 hr** | 953 | 99.0 | 343 | 95.9 |
| ^1^Linear or logistic regression models with generalized estimating equations or multinomial regression models with robust variance estimation were used to compare characteristics between groups. LSI, Living Standards Index calculated based on household assets and dwelling characteristics using principal components analysis. HFI, Household Food Insecurity estimated using a 9-item questionnaire collapsed into an index with possible scores ranging from 9 to 36 and categorized as none (HFI=9), mild (HFI>9 to HFI<16), and severe (HFI≥16). SSC, Secondary School Certificate. LAZ, length-for-age z-score; WLZ, weight-for-length z-score; WAZ, weight-for-age z-score. * P < 0.01, ** P < 0.05, *** P < 0.001. | | | | |
|  | | | | |

| **Supplemental Table 2.** Characteristics of household poultry, livestock, and fishpond ownership among infants enrolled in a longitudinal observational study nested within a trial providing a six-month protein intervention in rural Bangladesh (N=1467) | | | | | | | | | | |
| --- | --- | --- | --- | --- | --- | --- | --- | --- | --- | --- |
|  | | Number owned |  | Number owned by season | | |  | Ownership by gender | | |
|  |  |  |  | Winter  (Oct - Feb) | Summer  (Mar - May) | Monsoon  (Jun - Sep) |  | Female owned | Male owned | Female/ male co-owned |
| Type | *n* | Median  (25^th^, 75^th^ pctl) |  | Median  (25^th^, 75^th^ pctl) | Median  (25^th^, 75^th^ pctl) | Median  (25^th^, 75^th^ pctl) |  | % | % | % |
| Chicken | 1337 | 4 (2, 6) |  | 4 (2, 6) | 3 (1, 5) | 4 (2, 6) |  | 85.4 | 4.1 | 10.5 |
| Duck | 1335 | 3 (2, 5) |  | 3 (2, 6) | 2 (2, 3) | 3 (2, 5) |  | 85.2 | 3.6 | 11.3 |
| Cow/Buffalo | 1335 | 2 (1, 3) |  | 2 (1, 3) | 2 (1, 3) | 2 (1, 3) |  | 13.5 | 56.4 | 30.1 |
| Goat/Sheep | 1337 | 2 (1, 3) |  | 2 (1, 3) | 1 (1, 2) | 2 (1, 2) |  | 47.3 | 27.5 | 25.2 |
| Fishponds | 1323 | 1 (1, 1) |  | 1 (1, 1) | 1 (1, 1) | 1 (1, 1) |  | 3.3 | 81.6 | 15.1 |

| **Supplemental Table 3.** Associations between household animal and fishpond ownership and animal source food consumption observed among infants at three timepoints (6, 9, and 12 months of age) who were enrolled in a longitudinal observational study nested within a six-month protein supplementation trial in rural Bangladesh, complete case analysis (N=953)^1^ | | | | | | | | | | | |
| --- | --- | --- | --- | --- | --- | --- | --- | --- | --- | --- | --- |
|  | Seven-day frequency of  egg intake | |  | Seven-day frequency of  dairy intake | |  | Seven-day frequency of  meat intake | |  | Seven-day frequency of  fish intake | |
|  | Unadjusted | Adjusted |  | Unadjusted | Adjusted |  | Unadjusted | Adjusted |  | Unadjusted | Adjusted |
| Poultry ownership | IRR  (95% CI) | IRR  (95% CI) | Ownership of dairy-producing animals | IRR  (95% CI) | IRR  (95% CI) | Ownership of meat-producing animals | IRR  (95% CI) | IRR  (95% CI) | Fish pond ownership | IRR  (95% CI) | IRR  (95% CI) |
| No poultry | 1.00 | 1.00 | No animals | 1.00 | 1.00 | No animals | 1.00 | 1.00 | No ponds | 1.00 | 1.00 |
| Low  (1-3 poultry) | 1.16  (0.94, 1.42) | 1.21  (0.96, 1.52) | Low  (1 animal) | 1.40  (0.86, 2.27) | 1.42  (0.87, 2.32) | Low  (1-4 animals) | 1.07  (0.80, 1.42) | 1.05  (0.79, 1.41) | -- | -- | -- |
| Medium  (4-10 poultry) | 1.36  (1.12, 1.64)** | 1.40  (1.13, 1.74)** | Medium  (2-3 animals) | 1.94  (1.29, 2.91)** | 1.86  (1.23, 2.82)** | Medium  (5-11 animals) | 1.19  (0.90, 1.58) | 1.14  (0.86, 1.53) | Medium  (1 pond) | 1.08  (0.92, 1.27) | 1.06  (0.88, 1.28) |
| High  (≥11 poultry) | 1.65  (1.28, 2.12)*** | 1.73  (1.30, 2.30)*** | High  (≥4 animals) | 2.61  (1.63, 4.18)*** | 2.05  (1.26, 3.35)** | High  (≥12 animals) | 1.73  (1.27, 2.37)** | 1.49  (1.08, 2.04)** | High  (≥2 ponds) | 1.32  (0.95, 1.84) | 1.10  (0.75, 1.61) |
| ^1^Negative binomial regression models with random intercepts specified for infant and cluster were used to assess associations. Variables included in adjusted models were infant age and sex, maternal age, socioeconomic status, and season. Poultry included chickens and ducks; dairy-producing animals included cows, water buffalo, goats, and sheep; meat-producing animals include poultry, cattle, and goats/sheep. Egg intake included that of chicken and duck eggs; dairy included animal milk and yoghurt; meat included flesh meat and liver from poultry, cattle, and goats/sheep; fish included fresh and dried fish. IRR, incidence rate ratio. * P < 0.01, ** P < 0.05, *** P < 0.001. | | | | | | | | | | | |

| **Supplemental Table 4.** Associations between household animal and fishpond ownership and animal source food consumption among infants at three timepoints (6, 9, and 12 months of age) who were enrolled in a longitudinal observational study nested within a trial providing a six-month protein intervention in rural Bangladesh, stratified by maternal decision-making power, complete case analysis (N=953)**^1^** | | | | |
| --- | --- | --- | --- | --- |
|  | Frequency of intake in the seven days prior to interview | |  | IRR/IRR |
|  | Low  decision-making  power | High  decision-making  power |  |  |
| Animal/fishpond ownership | IRR (95% CI) | IRR (95% CI) |  |  |
| Poultry ownership |  |  |  |  |
| No poultry | 1.00 | 1.00 |  | - |
| Low (1-3 poultry) | 1.37 (0.97, 1.93)* | 1.09 (0.80, 1.49) |  | 0.80 (0.50, 1.27) |
| Medium (4-10 poultry) | 1.52 (1.10, 2.08)** | 1.32 (0.99, 1.75)* |  | 0.87 (0.57, 1.32) |
| High (≥11 poultry) | 1.46 (0.96, 2.23)* | 1.97 (1.35, 2.89)** |  | 1.35 (0.77, 2.38) |
| Ownership of dairy-producing animals |  |  |  |  |
| No animals | 1.00 | 1.00 |  | - |
| Low (1 animal) | 1.94 (0.93, 4.05)* | 1.11 (0.58, 2.12) |  | 0.57 (0.22, 1.53) |
| Medium (2-3 animals) | 2.01 (1.09, 3.71)** | 1.77 (1.02, 3.08)** |  | 0.88 (0.39, 2.00) |
| High (≥4 animals) | 3.20 (1.58, 6.50)** | 1.38 (0.72, 2.65) |  | 0.43 (0.17, 1.11)* |
| Ownership of meat-producing animals |  |  |  |  |
| No animals | 1.00 | 1.00 |  | - |
| Low (1-4 animals) | 1.03 (0.68, 1.57) | 1.07 (0.72, 1.60) |  | 1.04 (0.58, 1.85) |
| Medium (5-11 animals) | 1.18 (0.78, 1.78) | 1.12 (0.75, 1.65) |  | 0.95 (0.54, 1.66) |
| High (≥12 animals) | 1.42 (0.90, 2.24) | 1.54 (1.01, 2.37)** |  | 1.09 (0.59, 2.02) |
| Fishpond ownership |  |  |  |  |
| No ponds | 1.00 | 1.00 |  | - |
| Medium (1 pond) | 1.21 (0.92, 1.58) | 0.96 (0.75, 1.23) |  | 0.80 (0.56, 1.14) |
| High (≥2 ponds) | 1.43 (0.87, 2.35) | 0.80 (0.45, 1.42) |  | 0.56 (0.26, 1.19) |
| ^1^Negative binomial regression models with random intercepts for infant and cluster were specified. Each model was adjusted for infant age and sex, maternal age, socioeconomic status, and season. Low decision-making power was defined as a score from 0-8, and high as a score from 9-16. Poultry included chickens and ducks; dairy-producing animals included cows, water buffalo, goats, and sheep; meat-producing animals include poultry, cattle, and goats/sheep. Egg intake included that of chicken and duck eggs; dairy included animal milk and yoghurt; meat included flesh meat and liver from poultry, cattle, and goats/sheep; fish included fresh and dried fish. IRR, incidence rate ratio; IRR/IRR, ratio of incidence rate ratios. The IRR/IRR is an estimate testing for significance of effect measure modification on a multiplicative scale; it assessed if the magnitude of the measure of the association (IRR) between animal/fishpond ownership and animal source food consumption significantly differed between infants with mothers who have high decision-making power versus those with low decision-making power. If IRR/IRR>1.0, the magnitude of the rate of animal source food consumption between infants living in households with animals/fishponds versus those without animals/fishponds was greater among infants who have mothers with high decision-making power compared to those with low decision-making power; IRR/IRR<1.0, the magnitude was lower; IRR/IRR = 1.0, there was no difference in magnitude. * P < 0.01, ** P < 0.05, *** P < 0.001. | | | | |

**Supplemental Figure 1.** Participant flow diagram of a longitudinal observational study nested within a cluster-randomized controlled trial providing a six-month protein intervention to infants in rural Bangladesh. Eligibility was defined as infants born to women enrolled in an mHealth intervention trial who reached 3 months of age during an initial one-year enrollment period (September 2018 – September 2019). This observational study was restricted to infants residing in clusters randomized to the control arm of the trial.
